# Survival of HIV-infected patients with high-grade non-Hodgkin’s lymphomas: A retrospective study of experiences in Zimbabwe

**DOI:** 10.1371/journal.pone.0239344

**Published:** 2020-09-17

**Authors:** Maudy C. P. Manyau, Tinashe Mudzviti, Simbarashe Rusakaniko, Elson T. Mberi, Charles C. Maponga, Gene D. Morse

**Affiliations:** 1 School of Pharmacy, College of Health Sciences, University of Zimbabwe, Mount Pleasant, Harare, Zimbabwe; 2 Newlands Clinic, Highlands, Harare, Zimbabwe; 3 Department of Community Medicine, College of Health Sciences, University of Zimbabwe, Avondale, Harare, Zimbabwe; 4 Department of Hematology, College of Health Sciences, University of Zimbabwe, Avondale, Harare, Zimbabwe; 5 Center for Integrated Global Biomedical Sciences, University at Buffalo, Buffalo, New York, United States of America; 6 Translational Pharmacology Research Core, University at Buffalo, Buffalo, New York, United States of America; European Institute of Oncology, ITALY

## Abstract

**Background:**

Rituximab in combination with chemotherapy is now widely accepted as standard of care for AIDS-related lymphomas (ARLs) of B-cell origin. However, the clinical impact of rituximab in resource limited settings remains unknown. Different settings and patient heterogeneity may affect the effect of any given treatment. The study objectives were to determine if rituximab use was associated with improved 18-month overall survival (OS) of patients with ARLs and to identify correlates of 18-month OS.

**Methods:**

A retrospective review of medical records of adult HIV infected patients treated for high-grade large cell non-Hodgkin’s lymphoma with chemotherapy +/- rituximab between 2015–2017 was conducted. Vital status and disease progression/relapse at 18 months were determined. Survival functions were estimated using Kaplan-Meier methodology. Equality of survival functions were assessed using Log-rank tests and Cox regression analysis to identify risk factors for mortality.

**Results:**

One hundred and twenty-four eligible medical records were identified. This was a cohort of black Africans with a median age of 42 (IQR: 33–47) and a 57% male gender distribution. Overall survival at 6, 12 and 18 months for the population was 75.9%, 44.0% and 30.6% respectively. Over the study period, 72.6% of patients were diagnosed with disease progression/ relapse. There was a higher rate of rituximab use in patients who were treated at a private institution and those with medical insurance. Rituximab use was not associated with a reduction in 18-month mortality [adjusted hazard ratio (aHR)1.28, (95% CI 0.63–2.60)]. Risk factors for 18-month mortality were male gender [aHR 1.89, (95% CI 1.04–3.43)], age 40+ years [aHR 2.49, (1.33–4.67)], receipt of <3 chemotherapy cycles [aHR 2.48, (95% CI 1.33–4.60)] and low socioeconomic status [aHR 2.44, (95% CI 1.28–4.67)].

**Conclusions:**

Predictors of mortality were male gender, older age, low socioeconomic status and receipt of a less than half of the recommended number of chemotherapy cycles. Rituximab use was not associated with an improvement in 18-month OS in Zimbabwean patients with ARLs.

## Background

Evidence based medicine (EBM) makes use of the highest quality of evidence to inform clinical decision making. While the best quality of evidence is obtained from synthesis of literature [1], the argument for “real life” data to assess effectiveness of a treatment remains valid. AIDS Malignancy consortium (AMC) trials have provided conflicting results with respect to the benefit of rituximab. Phase III trial AMC010 which compared cyclophosphamide, doxorubicin, vincristine and prednisolone (CHOP) to CHOP plus rituximab (R-CHOP) did not find a statistically significant benefit in the R-CHOP arm [2]. Conversely a later phase II study, AMC034, which assessed timing of rituximab in patients receiving an etoposide containing infusional regimen found that earlier rituximab resulted in better complete response rates (CR), 73% vs 55%. However, this did not translate to improved survival [3]. Subsequently, a metanalysis of 15 prospective trials (only 1 phase III included) concluded that rituximab plus chemotherapy was superior to chemotherapy alone. The authors indicated that adjustments for confounders was not possible, and data from randomized controlled clinical trials (RCTs) should take precedence [4]. Patient level analysis of pooled data from 1546 patients found that the odds of CR and OS were higher in patients treated with rituximab. The study findings were said to provide level 2b evidence in support of rituximab as standard of care [5].

Literature on the survival of African patients with lymphoma is steadily increasing. However, these studies included a relatively small number of HIV-infected patients (8–75) [6–10]. Notwithstanding, cohort studies of lymphoma patients in southern Africa report 12-month overall survival (OS) rates of 40–45% regardless of the treatment used [6, 9]. The impact of chemo-immunotherapy has not been reported in the existing literature. Overall, the effectiveness of rituximab appears to be attenuated by HIV-infection, and/ or heterogeneity of patients encountered in routine clinical practice. A large Italian cohort which included HIV positive and negative patients found no survival benefit from rituximab in a cohort of diffuse large B-cell lymphoma (DLBCL) patients, adjusted hazard ratio (aHR) 0.94, (p = 0.65) [11]. Conversely, a Canadian population-based study which only included HIV-negative patients found that the survival benefit of rituximab was maintained [12]. There is a general paucity of literature for the use of rituximab for ARLs in routine clinical practice. Due to the often-prohibitive cost, use of rituximab in Zimbabwe is limited to affluent patients. Ideally, effective treatments should be available to all who need them.

Zimbabwe has an adult HIV prevalence of 13.8% [13]. Non-Hodgkin lymphomas (NHLs) contribute 7% to the annual cancer incidence [14], which is higher than the global NHL incidence of 2.8% [15]. Threats to the effectiveness of R-CHOP in the Zimbabwean ARL population include viremia and immunosuppression [16], advanced disease, poor health seeking behavior of patients and quality of care [17]. Due to the additional cost associated with add-on therapy with rituximab, it is important to determine the impact of the treatment in this patient population and environment. The study objectives were to determine if rituximab use was associated with improved 18-month OS of patients with ARLs and to identify correlates of 18-month OS.

## Methods

### General setting

The study was conducted in Zimbabwe which is a lower middle-income country [18]. Participants were recruited from 3 study sites which were a public referral treatment centre, a private physician and a private voluntary organisation (PVO).

### Study design

Retrospective review of medical records was used to collect clinical data from the study sites. Vital status of patients was determined from institutional records and the Zimbabwe National Cancer Registry (ZNCR).

### Patients

The study included all HIV-infected patients aged 18–59 years with a new diagnosis of high-grade large-cell lymphoma treated with CHOP-like chemotherapy with or without rituximab between January 2015 and December 2017. Patients were identified by International Classification of Diseases-10 (ICD10) codes for non-follicular NHL (C83). All the records were screened for a histological confirmation of high-grade large-cell NHL and a documentation of HIV-infection. Patients with Burkitt’s lymphoma, CD20-negative lymphomas and those who died before receiving NHL treatment were excluded. Patients receiving rituximab had a confirmed CD20+ lymphoma.

### Patient characteristics

Information on sociodemographic variables age, sex, presence of medical insurance place of residence and treatment institution were captured. Further data were captured for baseline clinical variables; date of diagnosis, Ann-Arbor clinical stage, B-symptoms, lactate dehydrogenase (LDH), presence of antiretroviral therapy (ART), duration of ART and CD4+ cell counts. Treatment related clinical variables which were captured included chemotherapy regimen, late initiation of NHL treatment (>30 days from diagnosis), delayed chemotherapy cycle (cycle interval >30 days) and the number of treatment cycles received.

### Patient follow-up and outcomes

Patients were retrospectively followed for 18-months from NHL diagnosis. Vital status at the end of the follow-up period, date of death and date of documented disease relapse/ or progression were recorded.

### Statistical analysis

The Wilcoxon rank sum tests were used to compare median baseline variables, and chi-square tests were used for categorical variables. Cox regression analysis was used to estimate the adjusted and unadjusted hazard ratios for mortality between the two treatment groups. Kaplan-Meier analysis was used to estimate survival, and the log rank test was used to test differences in survival curves between the groups. All analyses were done using a 2-sided 5% level of significance in Stata® version 13.0. Statistical significance was concluded based on a p-value less than 0.05.

To explore the potential relationship between overall survival and socioeconomic status, a proxy score was developed. The proxy had a potential maximum score of 3. Patients were scored on place of residence (urban = 1, peri-urban or rural = 0), medical insurance cover (yes = 1, no = 0), and institution (private physician = 1, public institution/PVO = 0). In our experience, patients managed in the public sector who can afford to purchase their own medications fare quite well. Patients with a total score of 1 would most likely be those residing in urban areas and have easier access to the highly centralised treatment centres and some form of income which enables them to afford medication. The patients with a total score of 0, are more likely to be those who live in more remote areas with less access to care, therefore, patients with the lowest socioeconomic status were those with a total score of 0.

According to *Bennet et al*, missing data above 10% is likely to result in biased estimates [19], therefore variables missing more than 10% were excluded from analyses. The pattern of missing data was assessed and found to be missing at random. Listwise deletion was used for data analysis.

### Ethical approvals

Ethical approvals were obtained from Joint Research and Ethics Committee of University of Zimbabwe (JREC/71/19), and the Medical Research Council of Zimbabwe (MRCZ/B/1767).

## Results

A total of 628 medical records were retrieved. Twenty-nine duplicate records were identified and removed. Of the remaining 599, 475 were excluded for the following reasons: HIV-negative status (n = 223), died before chemotherapy (n = 98), CD20-negative lymphoma (n = 45), unknown HIV status (n = 41), age ≥ 60 years (n = 33) no biopsy results (n = 28), Burkitt’s lymphoma (n = 7). A total of 124 eligible medical records were identified.

Patients lost to follow-up over the study period were 16, 24 and 26 at 6, 12 and 18 months respectively.

### Baseline characteristics

Ninety-seven of these patients received chemotherapy alone, and 27 received rituximab with chemotherapy. All of the patients were treated with CHOP-like regimens. Baseline sociodemographic, clinical, and treatment characteristics are shown in Table 1.

**Table 1:** Baseline sociodemographic clinical and treatment characteristics.

Variable	All	R-CHOP	CHOP	P
	N = 124	N = 27	N = 97	
**Sociodemographic Characteristics**				
**Males,** n (%)	71 (57.2)	14 (51.2)	57 (58.8)	0.521
**Age**, median years (IQR)	42 (33–47)	43 (33–48)	41 (32–47)	0.760
**Medical Insurance,** n (%)	46 (37.1)	18 (66.7)	28 (28.9)	^*^<0.001
^a^**Residential area,** n (%)				
Urban	40 (32.8)	19 (73.1)	21 (21.9)	^*^<0.001
Peri-urban	52 (42.6)	5 (19.2)	47 (49.0)	^*^0.007
Rural	30 (24.6)	2 (7.7)	28 (29.0)	^*^0.020
**Treatment institution,** n (%)				
Public hospital	91 (73.4)	5 (18.5)	86 (88.6)	^*^<0.001
PVO	15 (12.1)	9 (33.3)	6 (6.2)	^*^<0.001
Private physician	18 (14.5)	13 (48.2)	5 (5.2)	^*^<0.001
^a^ **Low socioeconomic status,** n (%)	58 (47.5)	4 (14.8)	54 (56.8)	^*^<0.001
**Diagnosis year,** n (%)				
2015	27 (21.8)	12 (44.4)	15 (15.5)	^*^0.001
2016	35 (28.2)	8 (29.6)	27 (27.8)	0.855
2017	62 (50.0)	7 (26.0)	55 (56.7)	^*^0.005
**Clinical Characteristics**				
^**b**^**Ann Arbor Stage,** n (%)				
I/II	14 (11.8)	2 (7.7)	12 (12.9)	0.466
III/IV	105 (88.2)	24 (92.3)	81 (87.1)	
^**c**^**B-Symptoms,** n (%)	87 (77.7)	20 (83.3)	67 (76.1)	0.453
^**d**^**LDH IU/ml,**				
Median (IQR)	733 (460–1445)	765 (489–1588)	727.5 (440–1370)	0.845
^a^**ART at diagnosis,** n (%)	90 (73.7)	23 (88.5)	67 (69.8)	0.055
^**e**^**Time on ART**, n (%)				
<6 months	45 (38.8)	7 (25.9)	38 (42.7)	0.117
≥ 6 months	71 (61.2)	20 (74.1)	51 (57.3)	
**CD4+ cells/mm**^**3**^, median (IQR)	173.5 (81–304)	172 (82.5–268)	173 (81–336)	0.651
**CD4+ cells/mm**^**3**^, n (%)				
<50	9 (7.3)	2 (7.4)	7 (7.2)	0.806
51–99	23 (18.5)	6 (22.2)	17 (17.5)	
≥100	92 (74.2)	19 (70.4)	73 (75.3)	
**Treatment characteristics**				
**Chemotherapy of cycles,** median (IQR)	5 (2–6)	6 (3–6)	5 (2–6)	0.144
**First cycle more than 30 days from diagnosis date,** n(%)	26 (21.3)	4 (14.8)	22 (23.2)	0.362
**Cycle delayed more than 7 days,** n (%)	52 (43.0)	10 (38.5)	42 (44.2)	0.667

*p-value < 0.05.

^a^ 2 missing values for CHOP group.

^b^ 5 missing values (4 CHOP and 1 R-CHOP).

^c^ 12 missing values (9 CHOP and 3 R-CHOP).

^d^ 37 missing values, (29 CHOP and 5 R-CHOP).

^e^ 8 missing values for CHOP.

The major differences in demographic variables between the two treatment groups were mainly factors related to socioeconomic variables. The odds of being treated by a private physician were 17 times higher in the rituximab group (OR 17.1, 95% CI 5.3–55.3). Similarly, the odds of having medical insurance and living in an urban area for the R-CHOP group were 5-fold (OR 4.93, 95% CI 1.81–13.87) and 10- fold (OR 9.69, 95% CI 3.28–30.49) those of the CHOP group respectively.

### Clinical outcomes

Median survival for the cohort was 11.2 months. The median survival for patients receiving R-CHOP was 11.2 months, and 11.7 months for those receiving CHOP. OS at 6, 12 and 18 months is displayed in Table 2. Hazard ratios are also presented below using CHOP as a reference.

**Table 2:** Overall survival of patients that received R-CHOP compared to those that received CHOP alone.

Percentage Surviving	All	R-CHOP	CHOP (reference)	HR	95% CI
**6-month N = 118, % (**95% CI**)**	75.9 (66.8–83.1)	80.8 (60.6–92.0)	74.4 (63.7–82.8)	1.17	0.44–3.14
**12-month N = 100, %** (95% CI)	44.0 (34.5–54.0)	50.0 (31.1–68.9)	41.9 (31.0–54.6)	1.07	0.57–2.00
**18-month N = 98,** % (95% CI)	30.6 (22.2–40.6)	38.4 (21.6–58.6)	27.8 (18.5–39.4)	1.08	0.62–1.91

**Fig 1:**
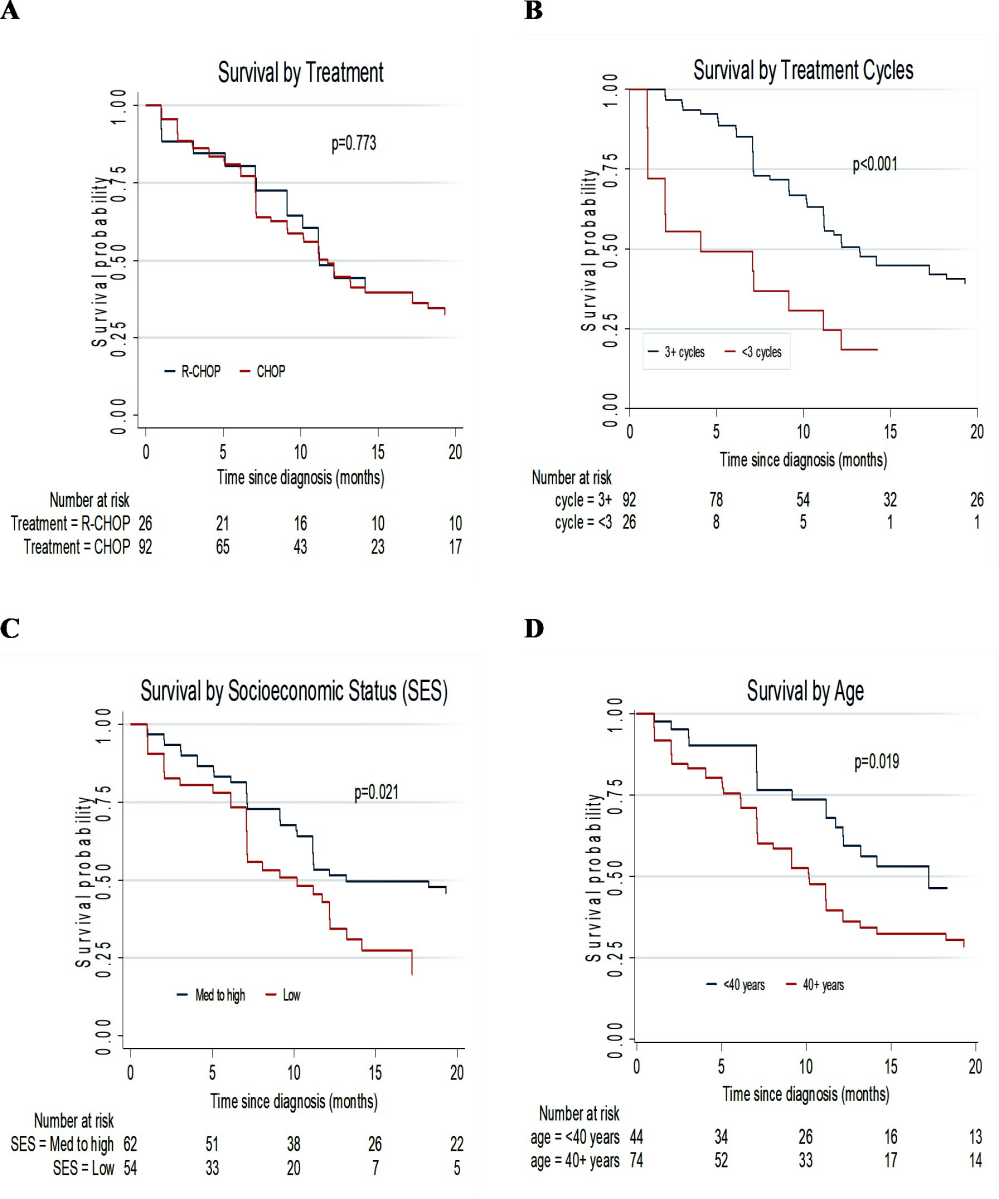
Kaplan-Meier plots comparing OS for patients with AIDS-related lymphomas according to: (A) NHL Treatment, (B) Number of cycles received, (C) Socioeconomic status and (D) Age <40 years and 40+ years.

There were no significant differences in OS between the two treatment groups as shown in Fig 1, Log-rank test p = 0.773. There were significant differences in survival functions for age ≥40 years (p = 0.019), receipt of <3 cycles of chemotherapy (p<0.001) and low socioeconomic status (p = 0.021).

Refer to Table 3 for adjusted and unadjusted hazard ratios (HR) of covariates.

**Table 3:** Cox regression analysis for the hazards of death at 18 months after diagnosis.

Covariate	Unadjusted HR	95% CI (p)	Adjusted HR	95% CI (p)
Rituximab	0.92	0.52–1.62 (0.776)	1.28	0.63–2.60 (0.495)
Male	1.58	0.95–2.62 (0.075)	1.89	**1.04–3.43 (0.036)**
Age 40+ years	1.86	1.09–3.17 (0.023)	2.49	**1.33–4.67 (0.005)**
Private physician	0.51	0.24–1.06 (0.072)	--	--
Medical insurance	0.69	0.42–1.14 (0.145)	--	--
Urban residence	0.95	0.58–1.57 (0.845)	--	--
Low socioeconomic status	1.78	1.07–2.90 (0.024)	2.44	**1.28–4.67 (0.007)**
CD4 < 100 cells/ mm^3^	1.32	0.78–2.24 (0.296)	--	--
ART at NHL diagnosis	1.07	0.62–1.84 (0.811)	--	--
ART treatment ≥ 6 months	0.80	0.48–1.34 (0.400)	0.71	0.39–1.29 (0.260)
Clinical stage III/IV	2.16	0.78–5.96 (0.137)	2.90	0.86–9.83 (0.086)
Chemo initiated >30 days after diagnosis	1.68	1.00–2.83 (0.050)	1.72	0.96–3.07 (0.069)
Chemo delayed >30 days	0.96	0.58–1.56 (0.856)	--	--
Receipt of < 3 cycles	2.99	1.69–5.31 (<0.001)	2.48	**1.33–4.60 (0.004)**

After adjusting for covariates, the risk factors for 18-month mortality included male gender, low socioeconomic status, receipt of <3 cycles of chemotherapy and age ≥ 40 years. The final predictive model had a χ^2^ value of 33.14, p<0.001.

Sixty-one (72.6% of evaluable patients, N = 84) were diagnosed with disease progression/ relapse. Sixty percent of patients receiving R-CHOP and 72.6% of patients receiving chemotherapy alone had disease progression, this was not statistically significant (p = 0.191). The hazard ratio for disease progression for patients receiving chemotherapy alone was 1.56 (95% CI 0.83–2.93). Of the patients with progressive disease, 18.5% were noted to have disease progression within 6 months of diagnosis, and the remaining 81.5% were diagnosed with disease progression after 6 months of diagnosis.

## Discussion

This is the largest study in southern Africa documenting survival of patients with ARLs, and the first to report on the clinical impact of R-CHOP for ARL in resource limited settings. This study examined the effects of rituximab as add-on therapy for high-grade non-Hodgkin’s lymphoma among people living with HIV (PLWH) in Zimbabwe. The main objective of the study was to determine if the 18-month OS was associated with rituximab use. R-CHOP was not associated with an improvement in the 18-month OS in this cohort [aHR of 1.28 (95% CI: 0.63–2.60)]. Disease progression was 60.0% for patients receiving R-CHOP, and 76.6% for patients receiving CHOP. This difference did not reach statistical significance.

There have been contradictory findings in the literature with respect to the efficacy of rituximab for ARLs. Studies which demonstrated that rituximab plus chemotherapy has a survival benefit for ARLs over chemotherapy alone, include phase II trials [20–22] and pooled analyses [4, 5]. A meta-analysis of 15 prospective clinical trials found that R-CHOP use was associated with an unadjusted odds ratio (OR) of 2.37 in 2-year OS when compared to CHOP [5].There was higher ART use in the rituximab arm, and adjusted OR were not presented. Similarly, in their pooled analysis, Barta et al found that the odds of achieving CR in patients who received rituximab was approximately 2.5-fold those of CHOP alone [5]. It should be noted that the lack of RCTs resulted in a disproportionate number of uncontrolled trials being included in these data synthesis studies. Barta et al indicated that their findings supported rituximab for ARL in the absence of definitive evidence [5].

Survival for this cohort was similar to that reported for ARL patients in southern Africa. The median survival for this cohort was 11.2 months which is comparable to median survival of 10.5 months observed in South Africa [8]. We observed a 12-month OS of 44.0%. This is comparable to 12-month OS of 44.7% for HIV infected lymphoma patients in Malawi [9], and 40% for patients from Botswana after a median follow up of 11.9 months [6]. In South Africa and Botswana R-CHOP is used as standard of care [6, 8]. Survival estimates from these countries as well as from a Malawian cohort that received CHOP only were similar to the observations made in the CHOP cohort from our study [9]. In general, survival rates in southern Africa are lower than those observed in Europe and North America [6, 11, 23]. This may be due to several factors, such as differential prognosis at diagnosis, quality of care and socioeconomic status of patients.

Risk factors for 18-month mortality in this cohort were male gender, low socioeconomic status, receipt of less than half of the recommended chemotherapy cycles (< 3) and age ≥40 years. No ART at diagnosis has been identified as a risk factor for mortality in African studies [7, 8]. A large European cohort found that patient on ART at NHL diagnosis had worse survival when compared to ART naïve patients [24]. The majority of patients in this cohort were on ART at diagnosis (73.4%), and there was a 39% risk of reduction in mortality for patients who had been on ART for at least 6 months, although this was not statistically significant (p = 0.260).

Gender appears to have a role in predicting survival of NHL patients. In the current study males had a 90% higher risk for 18-month mortality. Similar observations were made in a large Italian cohort, where females had an adjusted HR of 0.74 [11]. The generally accepted cut-off for age according to IPI is 60-years [25–28]. We found that age ≥ 40 years was associated with a 2.5-fold increase in the hazards of death, which is similar to a study that was conducted in Nigeria [10]. Larger studies have provided conflicting results on the prognostic relevance of age categories which are below 60 years of age. A large Italian cohort found a 10-year increase in age was associated with a 30% increase in the risk of death [11], conversely, another large European cohort, COHERE, did not confirm those findings [24].

The current study included a heterogenous population with respect to socioeconomic status. Low socioeconomic status was found to be associated with a 2.44-fold increase in the risk of death at 18-months, suggesting that health disparities exist within the population. In the US, NHL patients without private insurance had a 69% higher risk of mortality at 5-years [29]. Medical insurance alone did not predict survival. This may have been because there was no delineation between the types of packages. Currently, there is paucity of literature for health disparities that occur in resource limited settings. It has been proposed that if existing disparities can be identified, then disadvantaged groups can be recruited into clinical trials and cohort studies to improve access to care [30].

Threats to internal validity of the study included lack of adequate information on pathology and treatment response. The inclusion criteria were based on a histological diagnosis of high-grade large cell NHL, and CD20 positivity was only mandatory for the rituximab arm. Consequently, participants that received CHOP may have harboured other lymphoma subtypes such as plasmablastic variants which have poorer prognosis [31]. The lack of accurate information on treatment response led to the combining of disease relapse and progression. Separation of relapsed disease and progressive disease would have provided better insight into treatment responses. Additionally, accuracy of estimates for survival and disease progression/ recurrence may have been affected by patients who were lost to follow-up. The disease progression/ relapse of 72% obtained in this study was probably biased. This is because patients who feel unwell are more likely to remain in care versus those who are well. Despite these shortcomings, the survival estimates obtained in the current study were similar to existing literature.

While it is noted that this is a relatively small retrospective study, and does not provide definitive evidence with respect to the effectiveness of rituximab, it does add to the body of literature. From the literature, it is clear that the effects of rituximab are attenuated in PLWH. Prospective trials should assess the effects of immune dysregulation on response to rituximab, as this may further refine patient selection. Prospective pragmatic multi-centre trials in African patients with typical NHL disease presentation should be conducted. This will help unpack specific patient characteristics which affect treatment response.

## Conclusions

Rituximab did not confer a survival benefit in Zimbabwean patients with ARL. Gender, age, socioeconomic status and the number of cycles received predicted OS. The data suggest that health disparities exist in the Zimbabwean population.

## Supporting information

S1 File (DOCX)

S2 File (XLS)

## Abbreviations

aaIPI: age-adjusted International Prognostic Index
aHR: adjusted hazard ratio
ART: antiretroviral therapy
ARL: AIDS-related lymphoma
CHOP: cyclophosphamide/ doxorubicin/ vincristine/ prednisolone
CI: confidence interval
DLBCL: diffuse large B-cell lymphoma
EBM: evidence based medicine
HR: hazard ratio
LDH: lactate dehydrogenase
IQR: interquartile range
ICD10: International Classification of Diseases-10
NHL: non-Hodgkin lymphoma
OR: odds ratio
OS: overall survival
RCT: randomised controlled trial
R-CHOP: rituximab/ cyclophosphamide/ doxorubicin/ vincristine/ prednisolone
ZNCR: Zimbabwe National Cancer Registry
