# Supplementary material for: Survival of HIV-infected patients with high-grade non-Hodgkin’s lymphomas: A retrospective study of experiences in Zimbabwe
Source: PLoS One. 2020 Sep 17;15(9):e0239344. doi: 10.1371/journal.pone.0239344 (PMC7498086; doi:10.1371/journal.pone.0239344)
Supplement: S1 File — (DOCX) [file pone.0239344.s001.docx]

**Table A1 in S1 file: Shapiro-Wilk W test for normal data**

| Variable | P-value |
| --- | --- |
| Age | 0.001 |
| CD4 cell count | <0.001 |
| LDH | <0.001 |
| Cycles | 0.003 |

**Figure A1 in S1 File: Histogram of Age distribution**

**Figure A2 in S1 File: Histogram of baseline CD4+ cell count**

**Figure A3 in S1 file: Histogram of LDH distribution**

**Table B1 in S1 file: Socioeconomic status score distribution**

| **Socioeconomic score** | **Frequency (%)** |
| --- | --- |
| 0 | 58 (46.8) |
| 1 | 26 (29.0) |
| 2 | 21 (16.9) |
| 3 | 9 (7.2) |

**Figure B1 in S1 file: Kaplan-Meier survival curves by socioeconomic score**

Log rank-test Chi-square = 4.10, p=0.251

**Figure B2 in S1 file: Kaplan-Meier survival curves by socioeconomic score 0-1 vs 2-3**

Chi-square=0. 61 Log-rank=0.434

**Figure C1 in S1 file: Survival of patients 40+ years of age in each treatment group**

Log-rank p=0.979

**Figure C2 in S1 file: Survival of patients <40 years of age in each treatment group**

Log-rank p=0.355

**Figure C3 in S1 file: Survival of patients receiving at least 3 treatment cycles by treatment group**

Log-rank p=0.432

**Figure C4 in S1 file: Survival of patients receiving at less than 3 treatment cycles age by treatment group**

**Figure C5 in S1 file: Survival of patients with a low socioeconomic status by treatment**

**Figure C5 in S1 file: Survival of patients with a high (score=3) socioeconomic status by treatment**

**Analysis by Treatment**

| **R-CHOP** | **CHOP** |
| --- | --- |
| **** | **** |

**Figure D1 in S1 file: Kaplan-Meier survival curves by socioeconomic status stratified by treatment**

| **R-CHOP** | **CHOP** |
| --- | --- |
| **** | **** |

**Figure D2 in S1 file: Kaplan-Meier survival curves by age < or ≥ 40 years stratified by treatment**

| **R-CHOP** | **CHOP** |
| --- | --- |
| **** | **** |

**Figure D3 in S1 file: Kaplan-Meier survival curves by number of cycles received stratified by treatment**

**Figure D4 in S1 file: Kaplan-Meier survival curves by stage for R-CHOP group**

**Cox regression analysis**

**Table E1 in S1 file: Univariate hazards for 18 months mortality by treatment**

| **Covariate** | **Unadjusted HR** | | |
| --- | --- | --- | --- |
|  | **All (**95% CI; p) | **R-CHOP (**95% CI; p) | **CHOP** (95% CI; p) |
| Male | 1.58 (0.95-2.62; 0.075) | 2.21 (0.80-6.15; 0.128) | 1.38 (0.77-2.45; 0.275) |
| Age 40+ years | 1.86 (1.09-3.17; 0.023) | 2.64 (0.744-9.38; 0.133) | 1.66 (0.92-3.02; 0.095) |
| Low socioeconomic status | 1.77 (1.08-2.90; 0.024) | 10.38 (1.40-77.01; 0.022) | 1.77 (0.98-3.16; 0.056) |
| ART treatment ≥ 6 months | 0.80 (0.48-1.34; 0.400) | 0.69 (0.24-1.98; 0.489) | 0.86 (0.49-1.54; 0.616) |
| Clinical stage III/IV | 2.16 (0.78-5.96; 0.137) | 0.41 (0.09-1.86; 0.248) | 4.12 (1.00-17.04; 0.05) |
| Receipt of < 3 cycles | 2.99 (1.69-5.31; <0.001) | 4.92 (162-14.9; 0.005) | 2.55 (1.29-5.02; 0.007) |

**Table E2 in S1 file: Multivariate hazards for 18 months mortality by treatment**

| **Covariate** | **Adjusted HR** | | |
| --- | --- | --- | --- |
|  | **All** | **R-CHOP (**95% CI; p) | **CHOP** (95% CI; p) |
| Male | 1.98 (1.10-3.58; 0.23) | 1.58 (0.39-6.45; 0.521) | 2.01 (1.00-4.06; 0.49) |
| Age 40+ years | 2.58 (1.38-4.80; 0.003) | 2.04 (0.39-10.57; 0.394) | 2.59 (1.29-5.19; 0.007) |
| Low socioeconomic status | 2.12 (1.21-3.76; 0.006) | 13.13 (1.16-148.29; 0.037) | 2.00 (1.04-3.84; 0.037) |
| ART treatment ≥ 6 months | 0.71 (0.40-1.27; 0.248) | 0.30 (0.08-1.14; 0.078) | 0.80 (0.42-1.54; 0.514) |
| Clinical stage III/IV | 2.63 (0.80-8.61; 0.110) | 0.28 (0.04-1.64; 0.160) | 7.29 (0.98-54.30; 0.52) |
| Receipt of < 3 cycles | 2.56 (1.38-4.47; 0.003) | 3.76 (0.90-17.65; 0.093) | 2.43 (1.14-5.23; 0.022) |
